# Ecological Origins of Object Salience: Reward, Uncertainty, Aversiveness, and Novelty

**DOI:** 10.3389/fnins.2016.00378

**Published:** 2016-08-19

**Authors:** Ali Ghazizadeh, Whitney Griggs, Okihide Hikosaka

**Affiliations:** ^1^Laboratory of Sensorimotor Research, National Eye Institute, National Institutes of Health BethesdaBethesda, MD, USA; ^2^National Institute on Drug Abuse, National Institutes of HealthBaltimore, MD, USA

**Keywords:** object salience, reward, uncertainty, aversiveness, novelty

## Abstract

Among many objects around us, some are more salient than others (i.e., attract our attention automatically). Some objects may be inherently salient (e.g., brighter), while others may become salient by virtue of their ecological relevance through experience. However, the role of ecological experience in automatic attention has not been studied systematically. To address this question, we let subjects (macaque monkeys) view a large number of complex objects (>300), each experienced repeatedly (>5 days) with rewarding, aversive or no outcome association (mere-perceptual exposure). Test of salience was done on separate days using free viewing with no outcome. We found that gaze was biased among the objects from the outset, affecting saccades to objects or fixations within objects. When the outcome was rewarding, gaze preference was stronger (i.e., positive) for objects with larger or equal but uncertain rewards. The effects of aversive outcomes were variable. Gaze preference was positive for some outcome associations (e.g., airpuff), but negative for others (e.g., time-out), possibly due to differences in threat levels. Finally, novel objects attracted gaze, but mere perceptual exposure of objects reduced their salience (learned negative salience). Our results show that, in primates, object salience is strongly influenced by previous ecological experience and is supported by a large memory capacity. Owing to such high capacity for learned salience, the ability to rapidly choose important objects can grow during the entire life to promote biological fitness.

## Introduction

Attention can be used to actively search for and focus on something important (top-down attention). However, attention can also work passively in a more automatic fashion (e.g., bottom-up). For example, attention can be passively attracted by something bright, colorful or moving, which largely represents its physical features (physical salience). Attention is also automatically attracted to delicious foods (Nijs et al., 2010), erotic images (Lykins et al., 2006), faces (Hershler and Hochstein, 2005; Theeuwes and Van Der Stigchel, 2006), emotional expressions (Mogg and Bradley, 1999), and feared animals (Ohman et al., 2001), which represent ecological features. However, in such cases, the origin of salience (i.e., attentional bias) is often unclear. It can be present intrinsically, originate from past experience, or both combined (learned vs. intrinsic). In order to reveal the neural mechanisms of salience, it is critical to dissociate the different origins experimentally.

As the first step to address this question, we devised a learning procedure in which individual visual objects acquired different kinds and levels of salience through long-term experience. We let subjects (macaque monkeys) view many abstract fractal objects, each associated with a particular outcome or no outcome. Since monkeys had never seen these fractals, the ecological aspect of salience was created from scratch and fully controlled. Our experiments thus focused on “learned ecological salience.”

**Figure 1:**
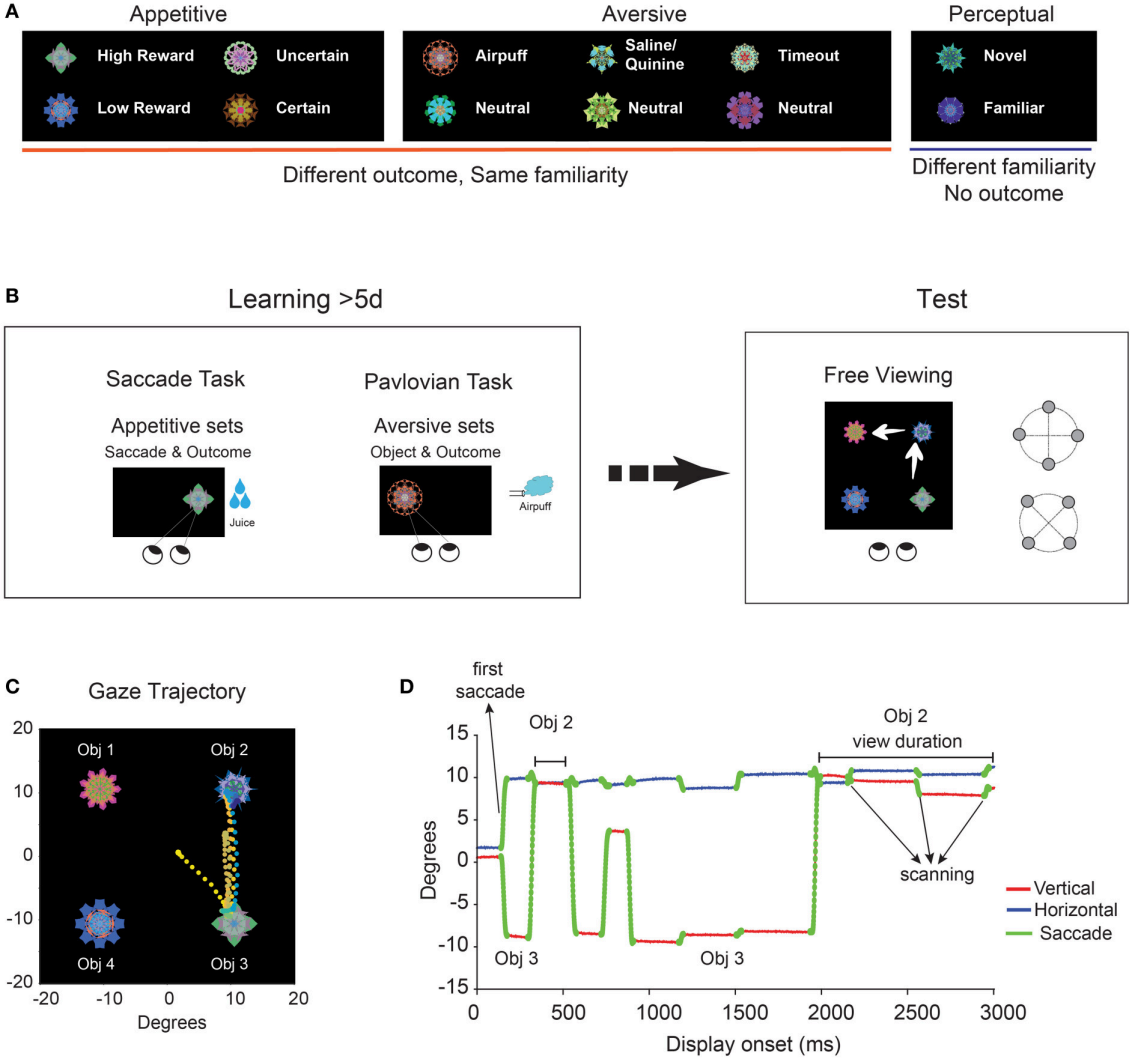
**Experimental paradigm and measures of learned salience. (A)** Subjects (macaque monkeys) viewed fractal objects in three ecological dimensions (appetitive, aversive, and perceptual) and their sub-dimensions. **(B)** Ecological learning and salience test. Procedures used for learning: Saccade task (appetitive), Pavlovian task (aversive), Free viewing (perceptual, not shown). The learning for each object lasted for more than 5 days. Free viewing in the absence of outcome was used to test the salience of individual objects in each sub-dimension. **(C)** Example of gaze trajectory composed of saccades and object fixations during a single free viewing trial (3 s). Eye position is shown by time-dependent color-coded dots (2 ms/sample, from orange: display onset to blue: display offset). **(D)** The time course of gaze during the same free viewing. Three metrics were used to quantify object salience: first saccade, object scanning and view duration (see **Figure 3**).

We considered three ecological dimensions: appetitive, aversive, and perceptual (Figure 1A). The appetitive dimension represents ecological outcomes that are desirable and promote approach (e.g., food seeking), while aversive dimension includes undesirable or dangerous outcomes that should be avoided (e.g., escaping from a predator). We further divided appetitive and aversive experiences depending on how the appetitive outcome was delivered and what kinds of aversive stimuli were delivered (sub-dimensions). For the third dimension, we perceptually exposed objects with no appetitive or aversive outcomes. We then contrasted these familiar objects with completely novel objects, which also did not predict any outcome to test the effect of mere-perceptual exposure.

We hypothesized that long-term experience along any of these dimensions should change object salience as measured by free gaze behavior. To test this hypothesis, we simply let subjects freely view the fractal objects following training. Orienting of gaze (eye movement) usually reflects orienting of attention (Rizzolatti et al., 1987; Motter and Belky, 1998), and can be called “overt attention” (Posner, 1980). Therefore, this free viewing procedure allows “free attention” to be measured. Biases in free viewing would reflect object salience in the absence of explicit task goals. To this end, we set two conditions for testing. First, objects and behavior did not predict any outcome during free viewing. Subjects were well-practiced in free viewing to know the absence of contingency. Second, the free viewing procedure was performed on separate days (>1 day) following the last learning session.

## Materials and methods

### General procedures

Four adult rhesus monkeys (*Macaca mulatta*) were used for the experiments (monkeys B, R, D male, and U female). All animal care and experimental procedures were approved by the National Eye Institute Animal Care and Use Committee and complied with the Public Health Service Policy on the humane care and use of laboratory animals. Monkeys were implanted with a head-post for fixation and scleral search coils to monitor eye movements prior to training in the tasks.

### Stimuli

For fractal stimuli, we used four point-symmetrical polygons that were overlaid around a common center such that smaller polygons were positioned more toward the front. The parameters that determined each polygon (size, edges, color, etc.) were chosen randomly (Miyashita et al., 1991; Yamamoto et al., 2012). Fractal sizes were on average ~7° × 7° but ranged from 5 to 10 degrees. Use of abstract fractals avoided previous perceptual exposures or appetitive/aversive associations that are common when using naturalistic stimuli and offered us complete control over object history for each subject. Furthermore, the fractal generation method allowed us to systematically create a large number of distinct fractal objects with recognizable variations in visual shapes and colors to test the memory capacity for previously experienced objects. The large number of fractals used per subject and their random assignment to various groups (below) also prevented possible differences in physical salience to systematically affect our findings. For face stimuli, we used 40 frontal conspecific monkey faces ~8° in size. Each monkey was familiarized with 8 faces (different for each monkey). The 32 remaining faces were novel and were used in novel vs. familiar free viewing (details below).

### Behavioral procedures

Behavioral tasks were controlled by a custom VC++ based software “Blip” (http://www.simonhong.org). Data acquisition and output control was performed using National Instruments NI-PCIe 6353. The monkeys sat in a primate chair with their head fixed facing a screen 30 cm in front of them. Stimuli generated by an active-matrix liquid crystal display projector (PJ550, ViewSonic) were rear-projected on the screen. Diluted apple juice was used as reward. Rewards amounts could be either small (0.08 and 0.1 ml for monkeys B, D and monkeys R, U, respectively) or large (0.21 and 0.35 ml for monkeys B, D and monkey R, U, respectively). Eye position was sampled at 1 kHz.

The behavioral procedure consisted of three tasks: saccade task, pavlovian task, and free viewing task. Saccade task was used to train object-reward and object-reward uncertainty association. Pavlovian task was used to train aversive sets that included rewarding, aversive, and neutral objects. Free viewing task was used to create object familiarity (fractal or face). Free viewing task was also used in all dimensions for measuring gaze bias between objects.

### Saccade task

We used an object-directed saccade task to train object value and risk associations for a set of fractals (Figure 1B, left). Trials started by the appearance of a central fixation dot. Following a variable fixation interval of 900 ± 200 ms, one of the fractals in the set appeared on the screen at one of the eight peripheral locations (eccentricity 15°). After an overlap period of 400 ms, fixation dot disappeared and the animal was required to make a saccade. Outcome was delivered after 500 ± 100 ms of fixating the fractal. This initiated inter-trial intervals (ITI) of 1250 ± 250 ms with a blank screen. All fractals were shown for the same duration after fixation regardless of reward outcome to ensure equal perceptual exposure. In the reward amount group (**Figure 3A**), outcome was small reward for half of fractals and large reward for the other half (4 low and 4 high reward fractals in a set). For the graded reward group (**Figure 3D**), outcome consisted of 5 grades of linearly increasing reward amounts from small to large (5 fractals in a set). In the reward uncertainty group (**Figure 4A**), outcome was medium reward for half of fractals and for the other half either small or large reward with equal probability (4 low and 4 high risk in a set). For the graded reward probability group (**Figure 4D**), outcome consisted of 5 grades of linearly increasing probabilities of receiving the large reward (0, 25, 50, 75, and 100%, 5 fractals in a set). Breaking fixation or a premature saccade to fractal resulted in an error tone and the same trial was repeated (<10% of trials). A correct trial was followed by another tone. Each training session consisted of 80 trials for a given set during which each fractal was presented 10 times (16 times for graded amount and probability sets) in a pseudorandom order.

### Pavlovian task

**Figure 2:**
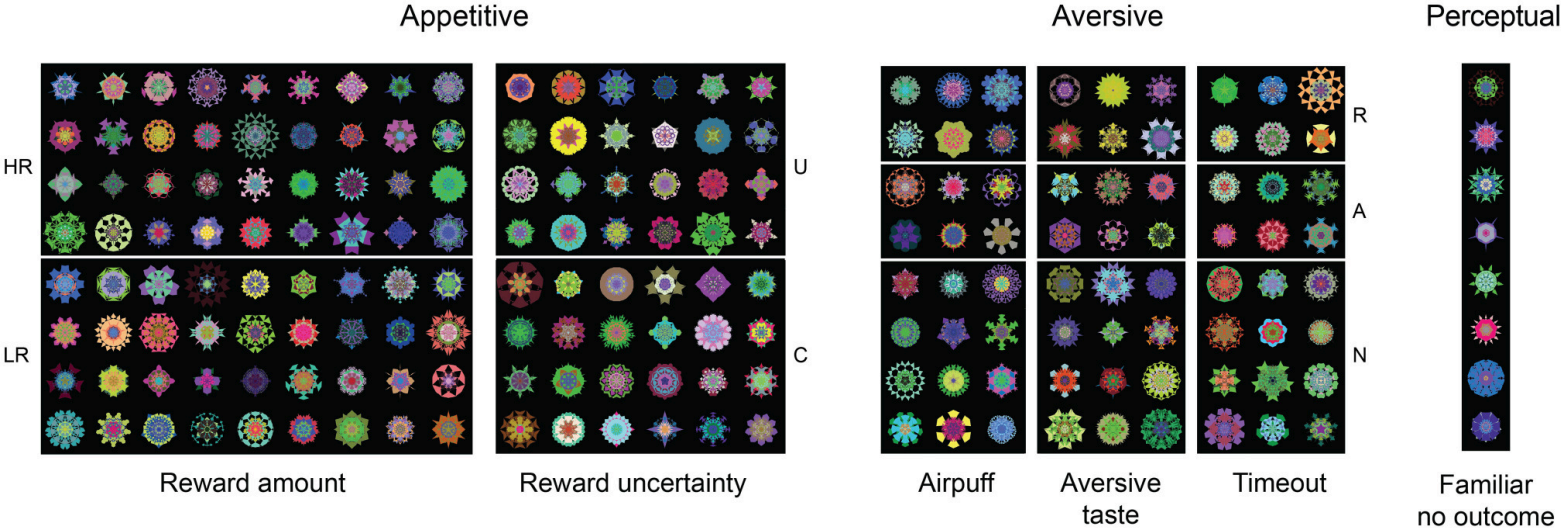
**Fractals experienced along various dimensions by a single subject (monkey U) with fractal numbers summarized in Table 1**. Not shown here are fractals used for reward gradient (Figure 3D, *n* = 15), reward probability (Figure 4D, *n* = 15), 45-trial free viewing (**Figure 8C**, *n* = 72), and novel fractals (**Figure 6A**, *n* = 32) as well as face stimuli (**Figure 6D**, *n* = 40). HR, high reward; LR, low reward; U, uncertain reward; C, certain reward; R, reward; A, aversive (airpuff, aversive taste, or timeout); N, neutral (no outcome). For perceptual dimension (right), eight fractals were experienced in free viewing with no outcome so that they became familiar objects. All subjects experienced the same number of fractals.

We used a pavlovian task to train aversive associations with a set of objects (Figure 1B, left). The main difference between this task and saccade task was that subjects were not required to fixate fractals to receive outcome during the pavlovian task. The rational for using the saccade task in reward and risk dimension was that it gives better control over perceptual exposure and foveation duration for each object. However, for aversive training, the requirement to fixate aversive objects would be impractical. In order to motivate our animals during aversive training, rewarding objects were also included in the aversive set along with aversive and neutral objects (Figure 2). All trials were preceded by an inter-trial interval (ITI) of 1250 ± 250 ms with a blank screen. After this, a fractal was shown randomly at one of 5 locations (4 radial, eccentricity 15° + center) for 500 ± 100 ms. Afterwards, the fractal was extinguished and the corresponding outcome was delivered. Neutral objects did not lead to any outcome. Reward objects resulted in a large reward. Airpuff objects resulted in 100 ms of 8-psi airpuff delivered to the right eye. The puff spout was positioned ~5 cm away from the temporal side of the eye. Tastant objects resulted in delivery of aversive taste whose volume was equal to the small reward. As aversive taste, normal saline was used for monkeys B and R and quinine (0.003 M Quinine HCl, Sigma-Aldrich) was used for monkeys D and U. Timeout objects resulted in an 8 s delay to start of the ITI. The ITI for timeout set was reduced to 750 ± 250 ms to enhance impact of this 8 s timeout. All aversive sets consisted of 8 fractals with 4 neutral, 2 aversive, and 2 rewarding objects (Figure 2, Table 1).

**Table 1:** **Number of objects used in various ecological dimensions per monkey**.

**Appetitive sets:**	**Aversive sets:**	**Perceptual sets:**
Reward high-low: 72 × 2^*^	Aversive: 18	Fractals: 44
High: 36 × 2	Airpuff: 6	Familiar: 8
Low: 36 × 2	Bad taste: 6	Novel: 36
Reward graded amount: 15	Time-out: 6	Faces: 40
Reward uncertainty: 48	Reward: 18	Familiar: 8
Uncertain: 24	Neutral: 36	Novel: 32
Certain: 24		
Reward graded probability: 15		
Total fractals: 338		Total faces: 40
Experienced: 302 Novel: 36		Experienced: 8 Novel: 32

**Figure 3:**
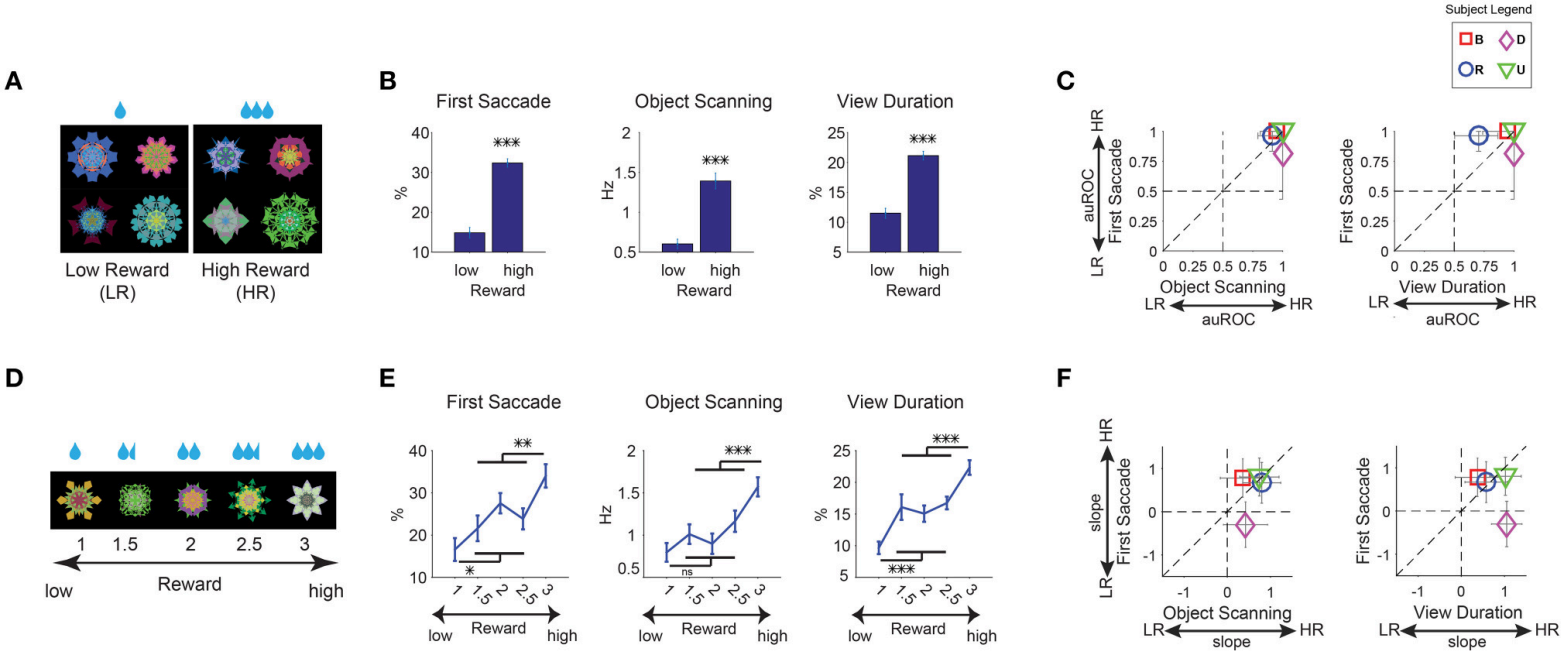
**Positive learned salience of appetitive objects: reward amount. (A–C)** Effects of large and small reward. **(A)** Example set of 8 objects. They were associated with low or high reward during learning, but not during salience test (free viewing). **(B)** Three measures of gaze bias. First Saccade: percentage of the first saccade directed from outside to inside object. Object Scanning: rate of within-object saccades. View Duration: total trial time during which gaze stayed inside object. See Figure 1D. These measures were averaged for high- and low-reward objects separately (*n* = 36 viewing sessions). **(C)** Gaze bias of individual subjects, shown by area under receiver operating characteristics (auROC). Error bar indicates 95% confidence interval. **(D)** Example set of 5 objects which were associated with a linear gradient of reward amount. **(E)** Three measures of gaze bias were averaged separately for the 5 different levels of reward amount. The statistical difference between highest and lowest reward levels compared with the three middle reward levels (Bonferroni *post-hoc*) are marked for significance (*n* = 24 viewing sessions). **(F)** Gaze bias of individual subjects, shown by the slope of a linear fit to gaze metrics as a function of reward amount. ^*^p < 0.05, ^**^p < 0.01, ^***^p < 0.001.

* *72 objects Figures 3A–C, 72 objects **Figures 8C,D***.

### Choice trials

To measure relative object value, choice trials between different fractal types within a set was included in separate sessions of saccade task or pavlovian task. Choice trials were similar to regular saccade task trials, except that two fractals were presented at the diametrically opposite positions. The animal was required to make a saccade to one of the targets and fixate it to get the outcome associated with that object. For the aversive sets, the animal was allowed to make multiple saccades before finally committing to one object within 3 s. This strategy was taken to give monkeys enough time to make their final decision and not be penalized for initial saccade.

### Free viewing task

Each free viewing session consisted of 15 trials with fractals chosen from one set of fractals. In any given trial, four fractals would be randomly chosen from a set (normally 8 objects except for graded amount set with 5 objects) and shown in one of the two spatial configurations (diamond or square) with 15 degrees eccentricity from the center of screen (Figure 1B, right). Location and identity of fractals shown in a trial would be chosen at random. For example, in the high/low reward free viewing, a given trial could have anywhere between 0 and 4 high reward objects shown in any of the four corners of an imaginary diamond or square around center. Fractals were displayed for 3 s during which the subjects could look at (or ignore) the displayed fractals. There was no behavioral outcome for free viewing behavior. After 3 s of viewing, the fractals would disappear. The initial eye location prior to display onset was unconstrained in order to create more contrast between free-viewing and the saccade task, where saccade after fixation was rewarded. Due to equal probability of objects appearing in a given location and the large number of trials per animal, the initial eye location could not affect our results systematically. Once fractals were turned off and following a delay of 600 ± 100 ms, a white fixation dot would appear in a one of nine random locations in the screen (center or eight radial directions). Monkeys were then required to make a saccade to the dot and maintain fixation (1 s) to obtain reward. This reward was not contingent on free viewing behavior and was included to maintain arousal. An ITI of 1250 ± 250 ms with a blank screen preceded next display onset with 4 fractals.

### Data analysis

**Figure 4:**
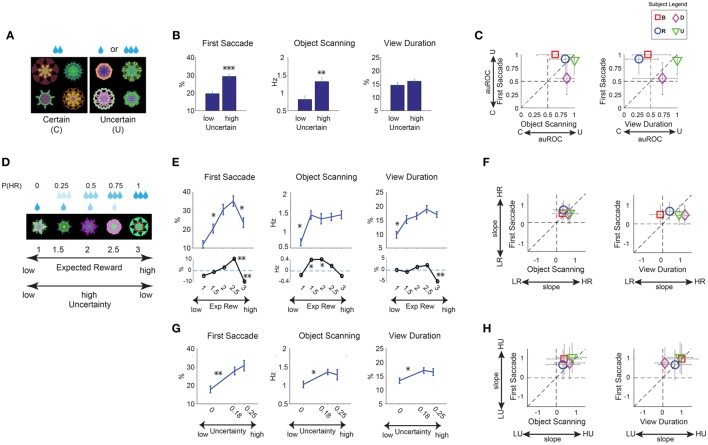
**Positive learned salience of appetitive objects: reward uncertainty. (A–C)** Same format as in Figures 3A–C for effects of reward uncertainty. (*n* = 24 viewing sessions). **(D)** Example set of 5 objects which were associated with a linear gradient of large reward probability, P(HR). **(E)** Three measures of gaze bias were averaged separately for the 5 different levels of reward probability (top plot). The statistical difference between reward probabilities (hsd *post-hoc*) are marked for significance (*n* = 24 viewing sessions). Subtraction of gaze bias in reward amount sets from reward probability (bottom plot, black lines) shows enhancement of salience by uncertainty for midlevel reward probabilities (significant differences from zero are marked). **(F)** Same format as Figure 3F but showing gaze bias slope as a function of reward probability for individual subjects. **(G)** Same format as **(E)** top but when objects were grouped by 3 different levels of reward variance (P(HR) = [0,1] with 0, P(HR) = [0.25,0.75] with 0.18 and P(HR) = 0.5 with 0.25 reward variance). The statistical differences between reward variances (hsd *post-hoc*) are marked for significance. **(H)** Same format as **(F)** but showing gaze bias slope for uncertainty levels for individual subjects. ^*^p < 0.05, ^**^p < 0.01, ^***^p < 0.001.

Data analysis and statistic tests were done using MATLAB 2014b using custom written software. Gaze locations were analyzed in an automated fashion and saccades (displacements >0.5°) vs. stationary periods were separated in a given trial (Figure 1D, example trial). Objects were considered to be fixated when gaze was within a 10 degrees window of their center with a stationary period present. Three gaze metrics were used to quantify object salience. (1) Percentage of first saccade to a given object following the display onset (chance level: 25% in our free viewing with 4 objects). (2) Object scanning rate: total number of saccades within an object over the viewing duration of that object. (3) View duration: total time spent viewing a given object divided by the total trial time of 3 s. For 2D plots of individual subject data (first saccade vs. scanning rate and first saccade vs. view duration), the effect of training on each metric was quantified using the area under the receiver operating characteristic (auROC). The auROC values for each measure and each subject was then plotted against each other (e.g., Figure 3C). For the graded reward or probability groups, the slopes of gaze metrics as a function of expected reward amount was calculated with positive slopes indicating increasing gaze bias as function of reward amount (Figures 3F,4F). For the reward probability group, the level of reward uncertainty (variance) was added as a second regressor (Figure 4H).

### Blink detection

Blinks were detected using the stereotypical deflections in the eye signals recorded using the eye coil signal. Blinks cause multiple peaks in the velocity profile and are detectable even during a saccade in both humans and monkeys (Collewijn et al., 1985; Goossens and Van Opstal, 2000). Detection of multiple peaks in close proximity of each other (20–100 ms, minimum peak height and prominence: 200° and 50°/sec, respectively) was taken to indicate presence of 1 blink. We validated this blink detection methodology in one session with simultaneous use of infrared camera for blink detection (EyeLink 1000 Plus). The specificity and sensitivity of our method were >90 and >80%, respectively. Thus, blink detection can be done with relatively high confidence using the eye coil data.

### Statistical test and significance levels

Two-way analysis of variance (ANOVA) with ecological factor (e.g., low or high reward) as fixed effect and subjects as random effect was done for all gaze metrics reported (e.g., Figure 3B). For trial by trial changes in gaze metrics, two-way ANOVA with ecological factor and trial number as fixed effects including the interaction term were used (**Figures 7, 8**). For dependence of novelty bias on display condition (1 novel/3 familiar etc), two-way ANOVA with ecological factor and display condition as fixed effects were used (**Figure 9**). *Post-hoc* tests were done using Tukey's “hsd” (honestly significant difference, unless stated otherwise). Error-bars in all plots show standard error of the mean (SEM), except for 2D plots of individual subject data (e.g., Figure 3C) where error-bars show 95% confidence interval. Significance thresholds for all tests in this study was *p* < 0.05. ^*^ < 0.05, ^**^ < 0.01, ^***^ < 0.001 (two-sided).

## Results

We studied ecological dimensions and sub-dimensions one at a time. For each monkey (*n* = 4 monkeys B, D, R, and U), different sets of fractals were used for different dimensions. Within a given dimension, fractals were randomly assigned to different values along that dimension (Figure 2, Table 1). Previous studies in our laboratory suggest that changing object salience may require multiple days (>5 days) of training and may rely on different neural mechanisms compared with short-term flexible object outcome pairing (Yasuda et al., 2012; Kim and Hikosaka, 2013; Kim et al., 2015). We thus used long-term learning procedures (>5 daily sessions, >50 times of viewing for each object) to accumulate many exposures in each dimension for each object (Figure 1B, left).

After the long-term learning period, we started examining the monkey's gaze bias among the fractal objects using a free viewing task (Figure 1B, right). In each trial, four fractal objects were chosen randomly and presented at symmetric positions for 3 s and the monkey looked around freely. After the objects appeared, the gaze quickly jumped onto one object (i.e., large saccade to object), fixated within the object, scanned it (i.e., small saccades within the object), and jumped to another object (Figures 1C,D). We therefore used various independent metrics to quantify the gaze dynamics for each object: (1) percentage of the first saccade to the object, (2) rate of scanning saccades within the object, and (3) percentage of viewing time for each object in a trial.

### Learned salience of appetitive objects

We performed two experiments to examine the effects of reward value and reward uncertainty (risk) separately.

#### Reward value

During the long-term learning, half of the fractals were associated with a small amount of apple juice (low value), while the other half were associated with a large amount of apple juice (high value). On each trial, the monkey made a saccade to the presented fractal object and kept fixating it until the reward was delivered (Figure 1B, saccade task). Therefore, the perceptual exposure was equal between the high- and low-valued objects. Each monkey experienced 9 sets of fractals (72 objects: 36 high-reward, 36 low-reward; Table 1).

On a separate day after the last learning session, gaze preference was tested in the free viewing task using a set of 8 objects (Figure 3A). Free viewing results showed a robust gaze preference for high-reward objects [Figure 3B, 9 sessions per monkey, *F*_(1, 67)_ > 89, *p* < 0.001 all metrics]. The first saccade after the object appearance was strongly biased toward a high-reward compared to low-reward object (First Saccade). High reward objects were scanned more frequently with small saccades (Object Scanning). Overall, high reward objects were viewed longer than low reward objects (View Duration). These results were common among all four subjects (Figure 3C).

We then asked whether different reward amounts determined different grades of salience. We used new fractal objects and divided them into five levels of linearly graded reward amounts (Figure 3D, example set). Each monkey experienced 3 sets of objects (15 objects, Table 1, not shown in Figure 2). Gaze preference tended to increase with the reward amount in all three metrics (Figure 3E, 6 sessions per monkey) and for all subjects (Figure 3F, positive slopes). The average gaze preference among the three intermediate reward amounts was lower and higher than the preference for the highest and lowest amounts, respectively [*F*_(1, 91)_ > 6 for all significant differences).

#### Reward uncertainty

During the long-term learning, half of the fractals were associated with a medium amount of apple juice (certain), while the other half were associated with a small or large amount of apple juice (uncertain; Figure 4A). Thus, the expected reward amount (i.e., value) was the same between the two groups. Each monkey experienced 6 sets of fractals (48 objects: 24 certain, 24 uncertain; Table 1) during the saccade task (Figure 1B).

Free viewing results showed gaze preference for uncertain objects (6 sessions per monkey). Statistical difference was present for the first saccade after the object appearance and scanning saccades within objects [Figure 4B, *F*_(1, 43)_ > 22]. Viewing duration showed no significant difference between uncertain and certain objects [*F*_(1, 43)_ = 2.25, *p* = 0.14], possibly due to larger inter-subject variability (Figure 4C). The gaze preference for uncertain objects was accompanied for choice preference for uncertain objects. When two objects (certain and uncertain objects) were presented in choice trials (see Section Materials and Methods), subjects tended to choose the uncertain object by making a saccade to it (>79% all monkeys) to receive the associated outcome. This behavior is consistent with previous reports of risk-seeking in monkeys (McCoy and Platt, 2005).

These results suggest that reward uncertainty increases object salience independent of the associated reward size. If so, uncertain objects with low-rewards may have a salience similar to high-reward objects. To test this hypothesis, we used new fractal objects associated with a linearly graded probability of small or large reward (Figure 4D, example set). Each monkey experienced 3 sets of objects (15 objects, Table 1, not shown in Figure 2). The expected reward values associated with these objects were linearly distributed, which was the same as the objects with the graded reward amounts (Figure 3D). But they were associated with reward uncertainty, unlike the amount group objects. The uncertainty was highest for the objects with equal probabilities (50%) of the large and small rewards.

Free viewing data (Figure 4E) showed higher salience for objects with higher reward probability as expected [main effect of probability *F*_(4, 112)_ > 8.6, *P* < 0.001] in all subjects (Figure 4F, positive slopes). However, the data suggested a boost in salience of objects with reward uncertainty (Figure 4E) compared to objects with no reward uncertainty (Figure 3E). This was confirmed by taking the difference between these two sets of data [Figure 4E bottom, *t*_(46)_ > 2 for significant differences]. This boost was more evident in the first saccade and object scanning, consistent with data in Figure 4B. Notably, the first saccade bias toward objects with 75% large reward was stronger than objects with 100% large reward (Figure 4E). To further quantity the effect of graded uncertainty on salience, we grouped the reward probability objects (Figure 4D) by reward variance instead of reward value (Figure 4E). The results indicate that uncertain objects had higher salience [Figure 4G, main effect of uncertainty *F*_(2, 114)_ > 4.1, *P* < 0.05] in all subjects (Figure 4H, positive slopes), although the effect was unclear between the two high levels of uncertainty (0.18 vs. 0.25). Thus in contrast with the salience of graded reward amount objects which showed clear difference between high and intermediate reward amounts (Figure 3E), the effect of uncertainty induced salience may be saturated at higher levels.

### Positive or negative learned salience of aversive objects

**Figure 5:**
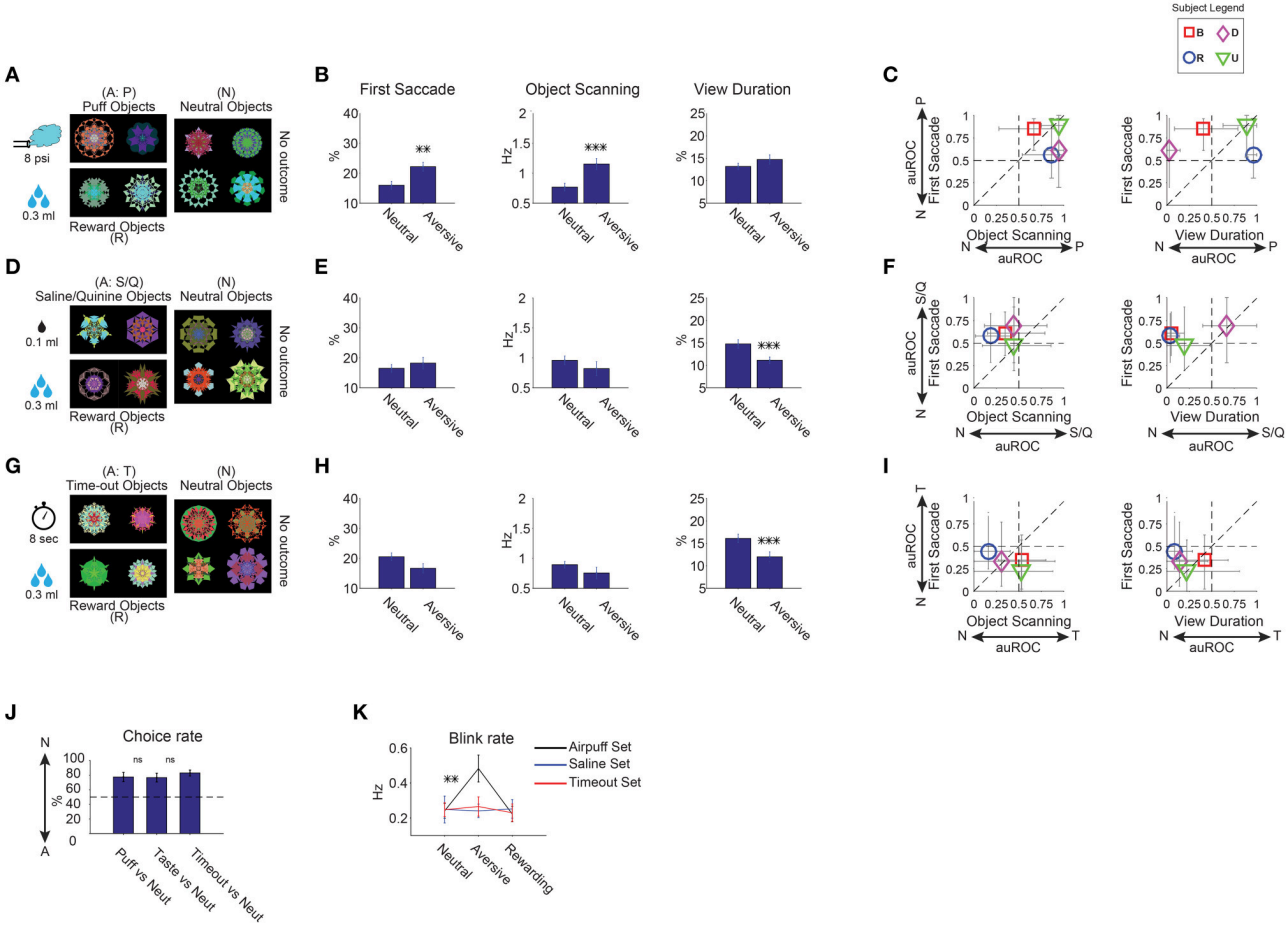
**Positive and negative learned salience of aversive objects. (A–C)** Effects of air puff. **(D–F)** Effects of aversive taste. **(G–I)** Effects of timeout. Same format as in Figures 3A–C (*n* = 30 free viewing sessions in each sub-dimension). Reward-associated objects were included during learning and test **(A,D,G)** but are not shown in **(B–C, E–F, H–I). (J)** Choice of neutral objects against aversive objects, shown separately for three sub-dimensions (*n* = 12 choice sessions). **(K)** Rate of blinking when viewing an object during free viewing in the three sub-dimensions. Blinking increased only when gaze was directed to airpuff-associated objects. Otherwise, the rate remained low, even when airpuff-associated objects were present, but outside gaze. ^**^p < 0.01, ^***^p < 0.001.

We examined three kinds of aversive outcomes independently: (1) airpuff directly delivered to the eye (Figure 5A), (2) aversive tastes (saline in monkeys B and R, quinine in monkeys D and U) delivered into the mouth (Figure 5D), and (3) 8 s time-out (Figure 5G). These outcomes have been used previously to study the short-term effects of aversiveness in decision making (Rolls, 2000; Matsumoto and Hikosaka, 2009; Morrison and Salzman, 2011; Leathers and Olson, 2012). In contrast, we studied the long-term effects of aversive association on object salience.

To study these three outcomes independently, fractals were divided into three groups. Each group contained three kinds of fractals which were associated with: (1) aversive outcome, (2) apple juice (reward), and (3) none (neutral). Rewarding objects were included to motivate monkeys during the learning. For the long-term training, we used a pavlovian task where continuous gaze on the presented object was not required to enable training with aversive outcomes (Figure 1B). Each monkey experienced 9 sets of fractals (72 objects)—3 sets for each aversive outcome (Table 1).

Free viewing results showed that the three aversive outcomes affected gaze preference differently (Figure 5, 6–9 sessions per monkey). The effect of airpuff (Figures 5A–C) was similar to the effect of reward value or reward uncertainty (Figures 3, 4). In particular, airpuff-associated objects induced the first saccade and within-object scanning saccades more often than neutral objects [*F*_(1, 55)_ > 10]. The airpuff effects on view duration were variable across the subjects and did not reach significance [Figures 5B,C, *F*_(1, 55)_ = 2.53 *p* = 0.11].

In contrast, largely opposite effects were induced by aversive taste (Figures 5D–F) and time-out (Figures 5G–I): these objects were viewed significantly shorter than neutral objects [*F*_(1, 55)_ > 19]. Within-object scanning saccades tended to be less frequent. Overall, the time-out effects were slightly stronger and more consistent across the subjects than the aversive taste effects (Figures 5E–F,H–I).

We assumed that all of the aversive outcomes used in our task were ecologically negative (i.e., monkeys disliked them), but this assumption might not be correct, which in turn may explain the differences in the gaze preference pattern (Figures 5A–I). To test this possibility, we tested preference for aversive objects using choice trials. An aversive and a neutral object were presented simultaneously and the monkey chose one of them resulting in an aversive or a null outcome (thus avoiding punishment). Results showed that the three groups of aversive objects were avoided in favor of neutral objects almost equally (Figure 5J), suggesting that they had roughly equal negative valences [*t*_(22)_ < 0.9, *p* > 0.17 pairwise comparisons].

Notably, we found an increase in eye blinking when the monkeys looked at airpuff-associated objects during free viewing [Figure 5K, *t*_(29)_ = 3.4]. The airpuff-related blinking occurred even though there was no outcome during free viewing. This did not occur when monkeys looked at other groups of fractals or other aversive objects (aversive taste, time-out, |t_29_| < 0.5 *p* > 0.7). Furthermore, the frequency of blinking for a given airpuff-associated object was positively correlated with the rate of first saccades to that object during free viewing (Pearson's *r* = 0.29 *p* = 0.02).

### Negative learned salience for familiar objects

**Figure 6:**
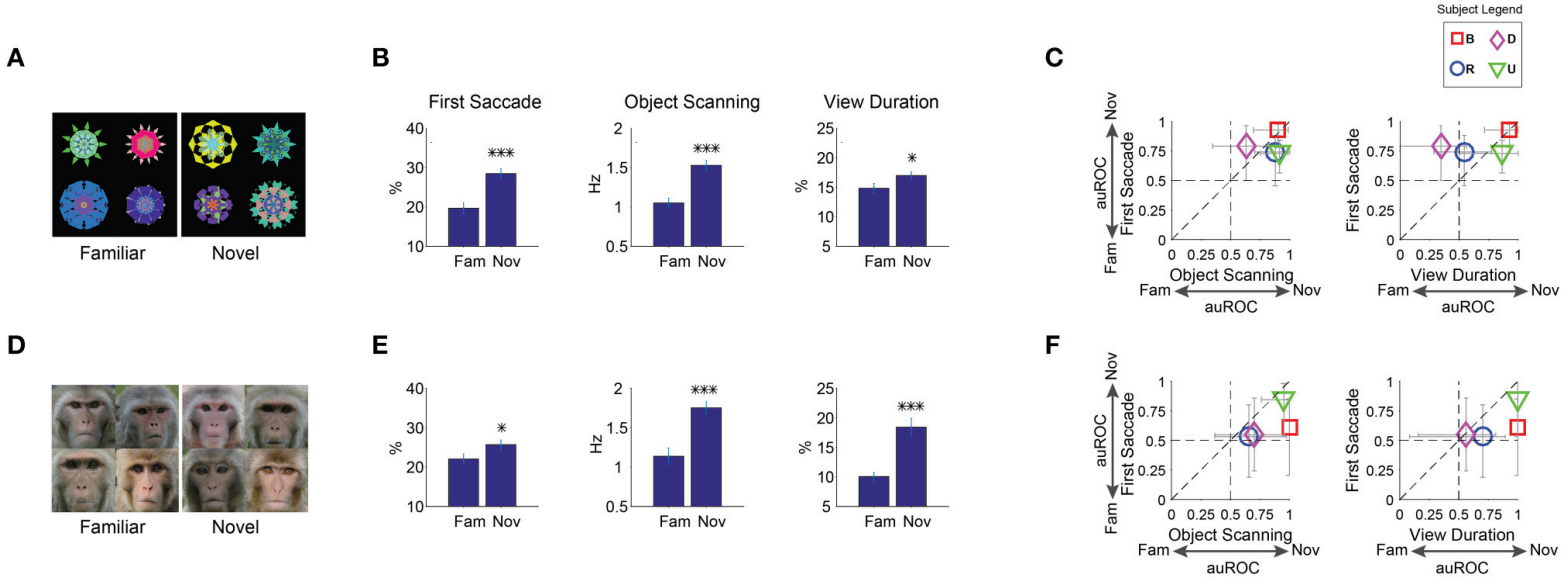
**Positive salience of novel objects (or, negative learned salience of familiar objects). (A–C)** Effects of familiarity on fractal objects (*n* = 36 viewing sessions). **(D–F)** Effects of familiarity on faces (*n* = 32). Same format as in Figures 3A–C. Before the salience test, each subject had viewed 8 fractals and 8 faces repeatedly in >5 free viewing sessions for familiarity training. Novel fractals and faces were viewed only in one test session. ^*^p < 0.05, ^***^p < 0.001.

To study the effect of mere perceptual exposure, we created two groups of fractals: novel and familiar. Monkeys had never observed the novel objects before salience testing (pure novelty). Familiar objects were created by letting the monkeys view them repeatedly (multiple free viewing sessions, >5 days) with no outcome (long-term familiarity). For the salience test, a set of 8 objects (4 familiar/4 novel) was presented in free viewing (Figure 6A, example set). Overall, each monkey saw 8 familiar and 36 novel fractals in 9 free viewing sessions (Table 1). When testing the effect of novelty, only one session was done per day for a given familiar object (last seen >1 day ago) to ensure equal short-term recency for familiar and novel objects.

Free viewing results showed that gaze was biased toward novel objects (Figures 6B,C). The effect of novelty was similar to the effects of reward value (Figures 3B,C). Statistical difference was present for the first saccade and within-object scanning saccades as well view duration [Figure 6B, *F*_(1, 67)_ > 6]. Overall, the novelty effect was somewhat weaker than the reward value effect. However, this may be due to the difference in their time courses, as explained below.

**Figure 7:**
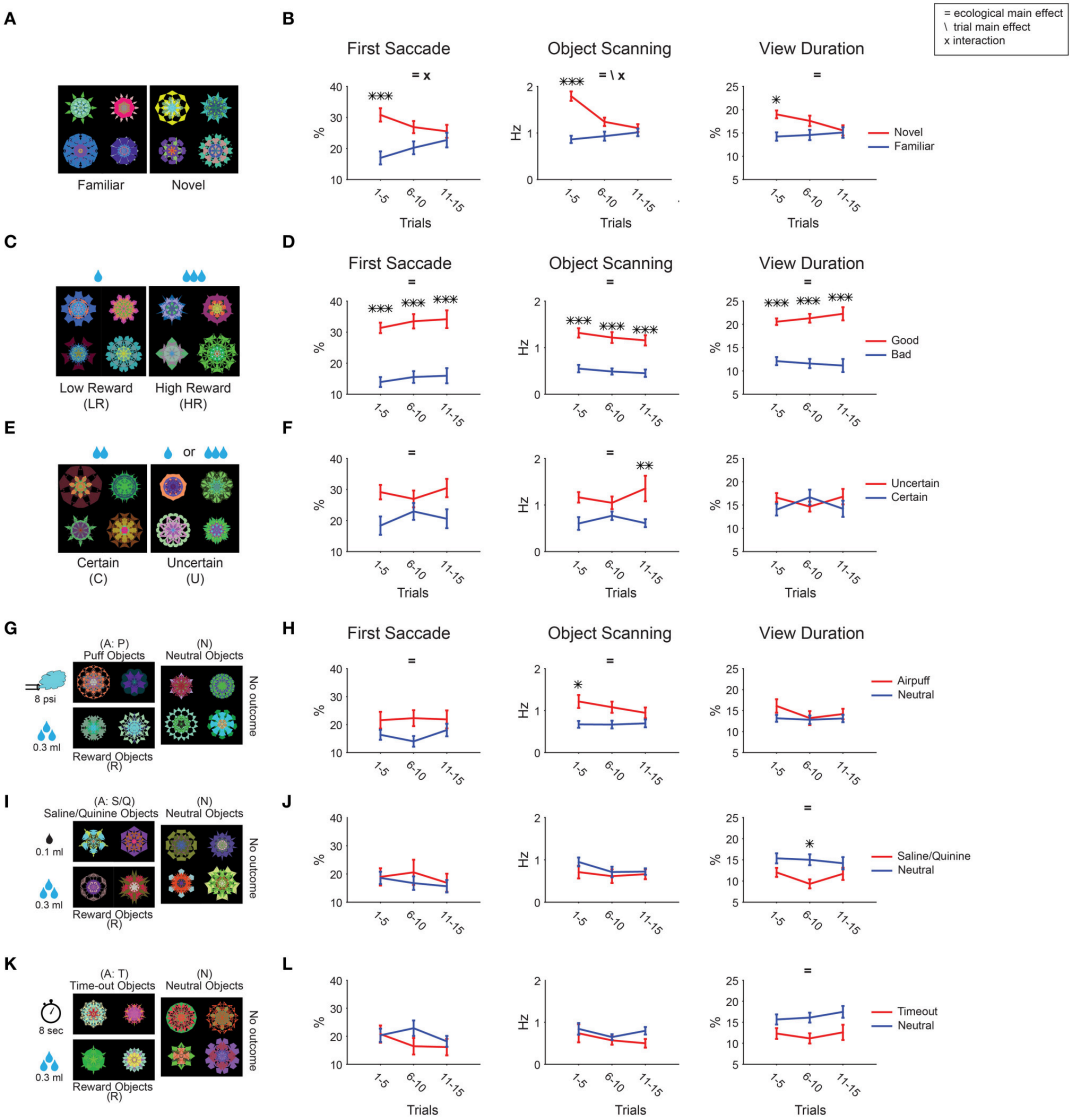
**Changes in object salience by repeated viewing with no outcome**. Gaze bias was measured during each session of free viewing, and averaged separately for the early (1–5 trials), middle (6–10 trials), and late (11–15 trials) periods. **(A,B)** Novel/familiar. **(C,D)** Low/high reward. **(E,F)** Certain/uncertain. **(G,H)** Airpuff/neutral. **(I,J)** Aversive taste/neutral. **(K,L)** Timeout/neutral. Main effect of ecological sub-dimension and trial number as well as the interaction between the two are marked. *Post-hoc* tests are marked with asterisks.

During a free viewing session, we used the same set of 8 objects that were randomly selected and shown in 15 trials. Thus, the same objects on average were shown 7–8 times within one test session. During each test session for novelty, gaze bias was initially present for all 3 metrics, but faded by the end of the session as the objects became increasingly familiar (Figures 7A,B). This phenomenon may be referred to as “negative learned salience for increasingly familiar objects.” In contrast, gaze bias for reward value remained unchanged during free viewing (Figures 7C,D). In the early part of the test session, gaze bias was comparable between novelty and reward value.

However, the effect of familiarity may also depend on the intrinsic properties of objects. In particular, it is suggested that faces may be treated differently from other geometric shapes in novelty preference (Park et al., 2010). Therefore, we let the monkey freely view familiar and novel faces (conspecific monkey faces) and studied whether gaze bias was differently affected by familiar and novel faces. Familiar faces were created using the same free viewing procedure for familiarity in fractals. On each trial in the salience test, a set of 8 faces (4 familiar/4 novel) was presented for free viewing (Figure 6D, example set). Overall, each monkey saw 8 familiar and 32 novel faces in 8 free viewing sessions (Table 1).

**Figure 8:**
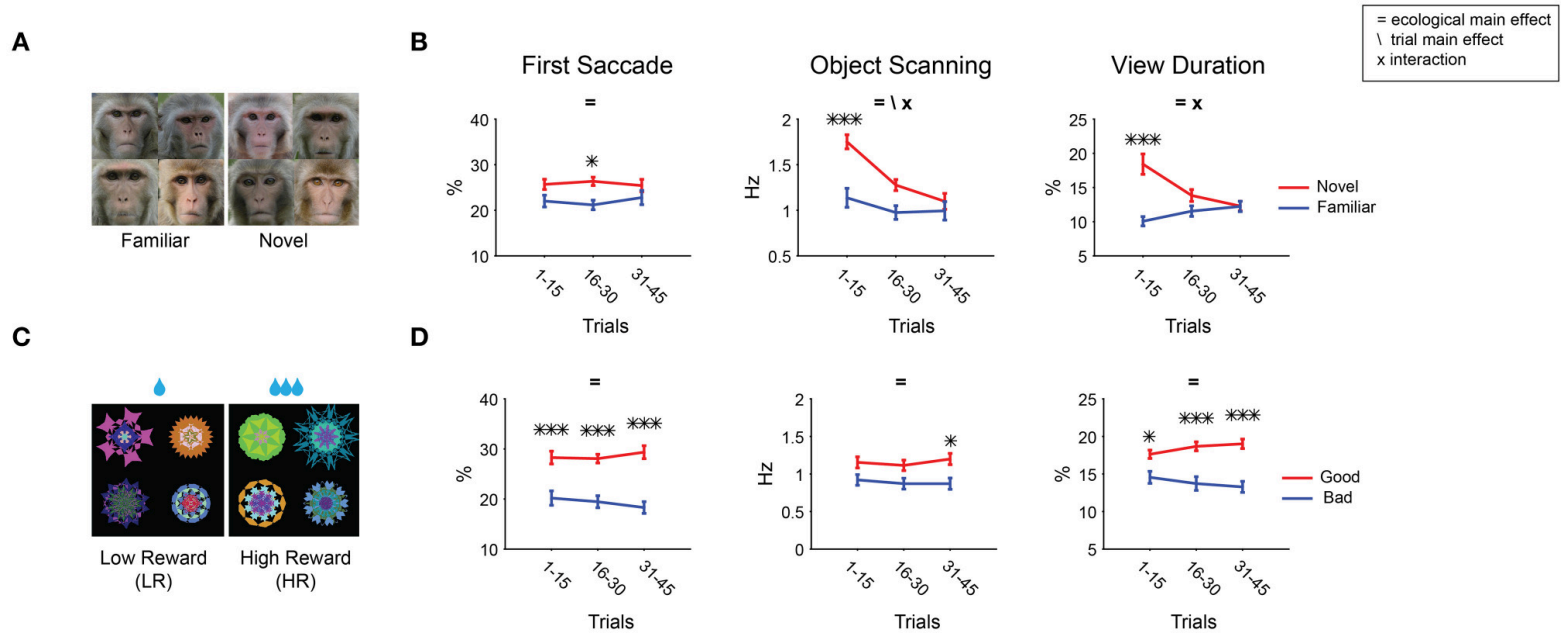
**Changes in object salience by longer repeated viewing with no outcome**. Free viewing test consisted of 45 trials (instead of 15 trials, Figure 7). **(A,B)** Novel/familiar faces. **(C,D)** Low/high reward fractals (additional 72 fractals, different from Figures 7C,D). Gaze bias for novel/familiar faces **(B)** declined more slowly than for novel/familiar fractals (Figure 7B). In contrast, gaze bias for low/high reward fractals never declined **(D)**. Main effect of ecological dimension and trial number as well as the interaction between the two are marked. *Post-hoc* tests are marked with asterisks.

Results show that gaze was attracted to novel faces in all three metrics (Figure 6E). The effects were stronger than that of novel fractals in viewing duration (Figure 6B). This difference is partly caused by their different time courses: Salience declined about three times slower for novel faces than for novel fractals (45 vs. 15 trials in Figures 8A,B,7A,B, respectively). Nonetheless, for both novel faces and fractals, salience disappeared eventually after repeated free viewing.

The decline of salience was unique to the novel-to-familiar transition [ecological × trial *F*_(2, 210)_ > 3.5 for fractal, *F*_(2, 184)_ > 4.5 for face; Figures 7, 8]. All other kinds of learned ecological salience we examined showed no significant change during one test session (Figures 7C–L, interactions *P* > 0.17). This is true even for 45 trial free viewing done with an additional group of value associated fractals (72 objects: 36 high-reward, 36 low-reward, Table 1, not shown in Figure 2; Figures 8C,D, interactions *P* > 0.16). We have previously shown that learned salience of rewarding objects remains virtually unchanged even when the test session (free viewing with no outcome) was repeated several times or was done many days after the long-term learning (Yasuda et al., 2012; Kim et al., 2015). These data indicate that novelty salience, which declines quickly, can eventually be replaced with other ecological dimensions of salience, which are retained stably.

**Figure 9:**
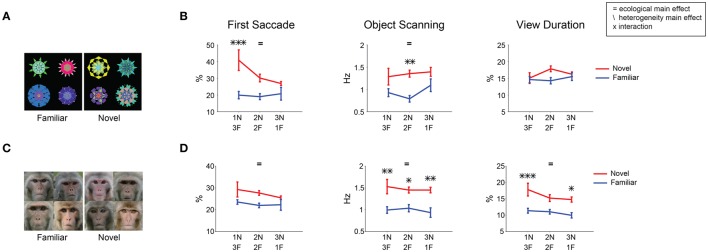
**Changes in novelty salience by varying its prevalence**. Free viewing trials were grouped into 3 display conditions: 1-novel/3-familiar, 2-novel/2-familiar, 3-novel/1-familiar objects, and analyzed for each gaze metric. **(A,B)** Novel/familiar fractals. **(C,D)** Novel/familiar faces. In both stimulus category and across the gaze metrics, novelty bias was observed regardless of condition (blue did not cross over red). Main effect of ecological factor and display condition as well as the interaction between the two are marked. *Post-hoc* tests are marked with asterisks.

While on average, novel objects were more salient than familiar objects, attention might be attracted to a familiar object if it is surrounded by novel objects. To examine this hypothesis, we analyzed free viewing data separately for three display conditions: 1-novel/3-familiar, 2-novel/2-familiar, and 3-novel/1-familiar objects (Figure 9). We found no condition where familiar objects were more salient than novel objects. For the first saccade, the novelty-based salience appears to be weaker in 3-novel/1-familiar condition with fractal objects (Figure 9B, left). This may be explained by the salience competition among 3 novel objects: the saccade to one novel object would be less frequent because it is also attracted to the other novel objects. Moreover, we found no significant change in the first saccade to familiar objects across the three conditions [one-way ANOVA: *F*_(2, 105)_ < 2.1 fractals, *F*_(2, 93)_ < 1 faces]. These results indicate that novel objects are more salient than familiar objects, regardless of novel object prevalence.

## Discussion

**Figure 10:**
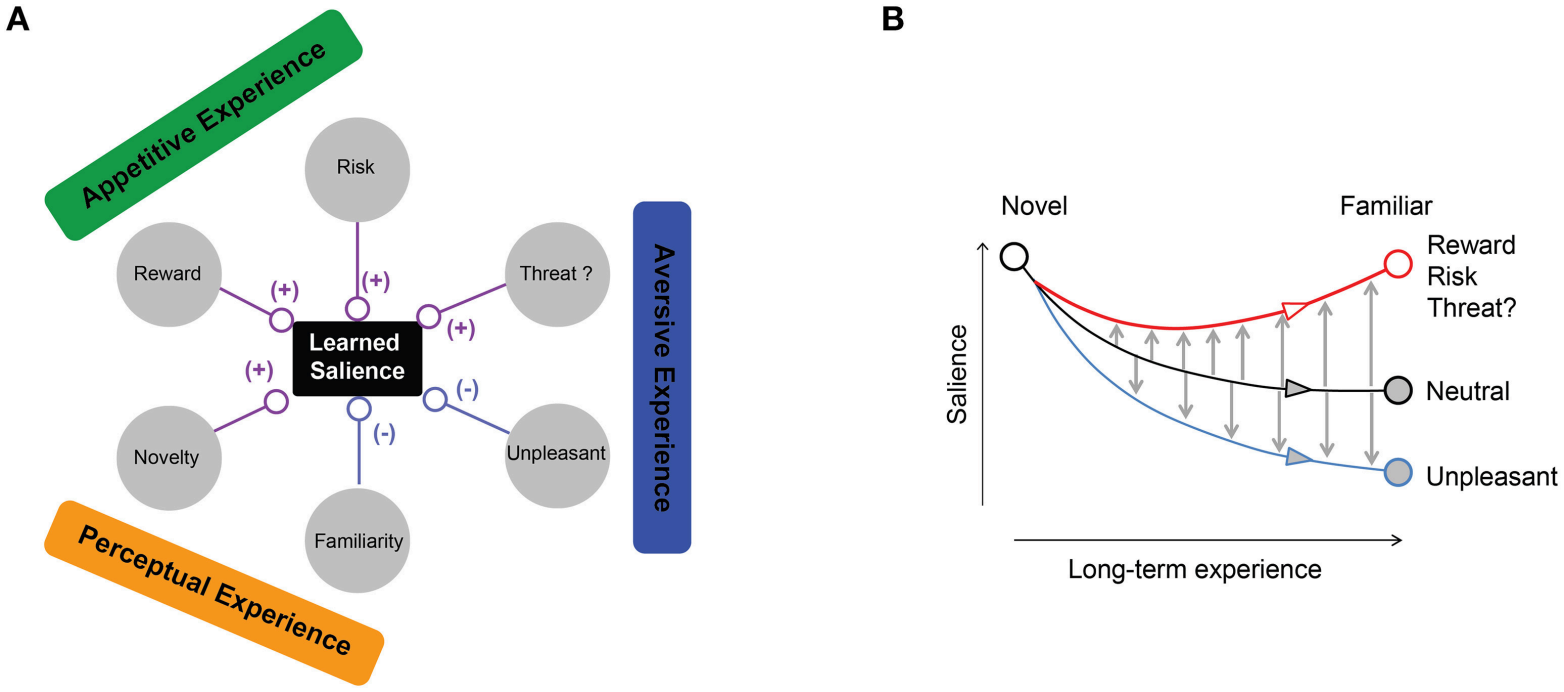
**Multiple ecological factors influencing objects learned salience. (A)** Appetitive dimension (reward amount or risk) enhances object salience. Aversive dimension enhances or suppresses object salience depending on the outcome type. Mere perceptual exposure suppresses object salience compared to novel objects. **(B)** Proposed dynamics of salience modification by ecological experience. Novel objects lose their salience through perceptual exposure (black line). Non-threatening but unpleasant outcomes further decrease object salience compared to neutral familiar objects. Rewarding, risky, or threatening outcomes counteract familiarity and enhance object salience.

Our study demonstrates that the salience of visual objects can change drastically after they were associated with appetitive and aversive experiences or by mere perceptual exposure. We conducted our experiments by using ecologically neutral objects (fractals) instead of ecologically enriched objects (e.g., guns or faces). The fractals were associated with particular outcomes while monkeys viewed them repeatedly. The monkeys then developed strong gaze biases among the fractals, thus proving “learned salience.” We also demonstrated that there are multiple ecological dimensions for learned salience, each affecting it positively or negatively (Figure 10), which suggests multiple neural mechanisms (discussed below). However, our results do not indicate that ecological salience is always acquired by learning. We will discuss each dimension separately below:

### Positive learned salience for rewarding or risky objects

Previous studies on learned salience mostly focused on the effect of reward amounts in humans (Della Libera and Chelazzi, 2009; Anderson et al., 2011; Theeuwes and Belopolsky, 2012) and monkeys (Yamamoto et al., 2013; Hikosaka et al., 2014). For example, task-performing human subjects are distracted by visual stimuli more strongly if they were previously used as target stimuli associated with higher monetary reward (Theeuwes and Belopolsky, 2012). However, the nature of other kinds of reward-related salience is less clear. Reward uncertainty (risk) influences choice (i.e., choose or avoid), but it has been unclear whether risk-associated objects attract attention automatically (i.e., salient). Our result shows a strong gaze/attentional bias for objects that were consistently associated with large reward or with large reward uncertainty (Figures 3, 4). This positive salience was also shown to be monotonically graded for objects with graded reward amount association (Figures 3D–F). We also saw increased salience for objects with a monotonically increasing reward probability. However, in this case the salience of objects with uncertain probabilities were boosted compared to objects with equal expected reward but no reward uncertainty (Figures 4D–F). Therefore, expected reward and uncertainty about reward can boost object salience independently.

The positive salience of risk-associated objects in our study was concurrent with risk seeking behavior during choice trials in monkeys. However, risk seeking in water motivated monkeys can be modified or even reversed (becoming risk aversion) by various factors such as thirst level, reward magnitudes and inter-trial interval (Hayden and Platt, 2007; Yamada et al., 2013; Stauffer et al., 2014). Whether such changes in risk seeking affect the learned salience of risky objects is an intriguing question that needs to be addressed in future.

### Positive learned salience of threatening objects

Aversiveness has various features and there are various ways to deal with it (e.g., ignore, escape, attack; Azrin et al., 1967). In fact, we found a difference between aversive objects in the way they engage our attention. We found that while airpuff associated objects were attended more than neutral objects, the opposite was true for aversive tastent or time-out objects (positive or negative salience, Figures 5A–I), despite their equivalent negative valence (Figure 5J). One possible explanation is that airpuff objects may be more threatening than other aversive objects such as time-out objects, which are merely unpleasant. The threatening quality of airpuff may be related to its role in startle response potentiation (Grillon and Ameli, 1998). We also found enhanced blinking during viewing of airpuff objects (Figure 5K). Blinking is known to be enhanced for threatening, but not disgusting, visual stimuli (Balaban and Taussig, 1994) and its strength as a conditioned response to airpuff is known to correlate with physiological responses to threat stimuli (Runquist and Ross, 1959). On the other hand, salience of time-out objects, which were unpleasant but not threatening, was negative. This result is consistent with enhanced attention to threatening but not sad faces reported in humans (Bradley et al., 2000; Sylvester et al., 2016). However, there may be different interpretations. For instance, airpuff generates fast sensory signals which are likely to induce fast reactions, possibly regardless of emotional outcomes. Further experiments are required to test such alternative explanations.

Note that, while in the case of ecologically enriched objects (e.g., threatening faces), the origin of positive salience (intrinsic or learned) is not clear, our results clearly show that the positive salience for aversive objects can be acquired through learning: initially neutral objects became salient if the monkeys experienced them in association with airpuff. Theeuwes and colleagues showed that a similar learning effect of threat-induced salience occurs in humans (Notebaert et al., 2013; Schmidt et al., 2015). However, intrinsic salience could also exist, which remains to be studied.

### Negative learned salience of familiar and unpleasant objects

Our results are consistent with a common concept that novel objects attract attention more than familiar objects (Johnston et al., 1990; Snyder et al., 2008; Yang et al., 2009). Novel objects attracted gaze more strongly than familiar objects using various gaze metrics and independent of familiar objects prevalence on the display (Figures 6, 9). Note, that in most previous studies, familiarity is established on a relatively short time scale within a given test session. We, on the other hand, focused on the long-term familiarity with objects that were seen many times over multiple days prior to testing (>5). Furthermore, our novel objects were completely new, never before seen by the subjects. Indeed, while we observed strong attentional bias to purely novel objects compared with purely familiar objects on the first saccade, the opposite pattern was observed when familiarity was established on a shorter time scale (Snyder et al., 2008). Novelty and familiarity can also be defined by recency of object experience (Wilson and Rolls, 1993; Mishkin and Murray, 1994). In this usage, novelty/familiarity depends on short term visual memory and may engage separate neural populations (see neural discussion). Importantly, we controlled for the effect of recency in our experiment by (1) having novel and familiar objects appearing with equal probability during the free viewing test and (2) using a familiar object only in one free viewing block in a given day. This ensured that novel and familiar objects had equivalent recency (short term experience) in our experiment.

Novelty-induced salience may appear different from reward- or threat-induced salience, because novelty is not created by learning. However, novelty is the opposite of familiarity that is created by learning. We show that novelty-induced positive salience is observed as the result of familiarity-induced negative salience (Figures 7, 8), similar to a concept proposed by Zelinsky and colleagues (Yang et al., 2009). Notably, the learning speed of the familiarity-induced negative salience was slower when faces (Figure 8) were presented instead of fractals (Figure 7). This may be due to the intrinsic salience of faces counteracting the development of negative salience due to familiarity.

Negative salience also developed for certain aversive outcomes (Figures 5D–I). Importantly, these aversive objects were less salient than familiar neutral objects, suggesting that the learned negative salience is acquired by two separate mechanisms related to familiarity and unpleasantness (Figure 10B). These negative salience mechanisms are important, because they let us ignore many objects that are familiar neutral or unpleasant, so that attention is accurately directed to ecologically salient objects.

### Novelty or familiarity preference: dissociating valence and salience

Previous studies have produced conflicting results with evidence for both familiarity (Zajonc, 1968; Berlyne, 1970) and novelty preference (Fantz, 1964; Johnston et al., 1990). However, studies of familiarity preference often measured subjective valuation (valence) of objects (Zajonc, 1968; Berlyne, 1970; Park et al., 2010) rather than their salience (i.e., ability to attract attention). In particular, Park et al. (2010) reported novelty preference for geometric shapes, but familiarity preference for faces. In contrast, we observed strong novelty salience for both fractal shapes and faces. This suggests a dissociation between attention to and valuation of stimuli. Such a dissociation between valence and salience resembles the dissociability of emotions along valence-arousal axes (Russell, 1980; Lang et al., 1993). Indeed, separability of valence and salience can explain the positive salience of airpuff objects during free viewing despite their negative valence.

### Object viewing and scanning: starting point of action and cognition

Sometimes, we need to examine an object (i.e., cognition) before preparing for an action. This occurs while gaze is fixed on or scanning the object (Yarbus et al., 1967; Just and Carpenter, 1976). Our data showed that more salient objects were fixated longer and scanned more frequently, suggesting that salience may support cognition. Encountering valuable, uncertain or threatening objects, we may need to decide as quickly as possible to reach, escape from, or attack them. Indeed, these objects had high levels of salience. In contrast, it is better to ignore or make no action to familiar but valueless objects or non-threatening aversive (unpleasant) objects. These objects had low levels of salience (Figure 9A).

The salience-action/cognition relationship may also apply to the novelty-based salience. The outcome of a novel object is uncertain and unknown. Moreover, it is unknown how variable the outcome can be (ambiguity), unlike reward uncertainty (risk). Therefore, we need to find the novel object quickly to get ready for a variety of actions.

### Vision, attention, and memory for ecological salience

Our data points to important features that characterize learned ecological salience:

1. *Peripheral vision*: According to our data, ecologically salient objects attracted the first saccade before any direct object fixation was available. This means that fractal objects presented in the periphery must be identified among many others for the saccade to aim at a salient object. Without such a mechanism, saccades would occur randomly across objects. Thus, peripheral vision was sufficient for complex object identification in our task despite limited visual resolution (Loschky et al., 2005). However, it remains to be tested how robustly our objects can be identified by peripheral vision against changes in stimulus size, eccentricity and density (Strasburger et al., 2011).
2. *Automatic attention*: During our testing procedure (i.e., free viewing), no contingent outcome was delivered (unlike during the learning), yet the monkey's attention/gaze continued to be automatically drawn to certain objects. Several findings in our experiment support that this gaze bias was maintained by a passive rather than an active top-down mechanism: (1) gaze bias toward high-reward objects was sustained with no sign of reduction during a long session of free viewing (45 trials), even though there was no reward outcome (Figure 8), (2) gaze was biased toward airpuff-associated objects, even though they were avoided during choice (Figure 5), (3) gaze bias occurred among faces (novel vs. familiar), even though they had never been associated with rewarding or aversive outcome (Figure 6), and (4) the bias was present in the first saccade evoked by free viewing onset in all dimensions tested. However, this does not exclude the possibility that deliberate cognitive strategies (such as reward seeking) may have contributed to gaze bias as well.
3. *High capacity memory*: Previous studies that have looked at learned salience used a limited number of simple objects (Anderson et al., 2011; Theeuwes and Belopolsky, 2012; Schmidt et al., 2015). In our experiments, each monkey viewed many complex objects (*n* = 302 appetitive, aversive and familiar, Table 1), each of which was associated with an ecological outcome (or no outcome), yet gaze bias occurred differently for these objects. This means that the underlying neural mechanism has a high capacity memory. In real life, we are surrounded by many complex objects, yet their ecological salience can be maintained by such high capacity memory to orient us toward salient objects and ignore others.

### Neural mechanisms of learned ecological salience

The diversity of experiences which result in learned ecological salience suggests different neural mechanisms might be involved. We discuss them separately below:

#### Reward value

Studies from our laboratory suggest that the caudal part of the basal ganglia underlies reward-value based salience (Yasuda et al., 2012; Kim and Hikosaka, 2013; Yamamoto et al., 2013). The responsible neuronal circuit is composed of: the tail of the caudate nucleus (CDt), the caudal-dorsal-lateral part of the substantia nigra pars reticulata (cdlSNr), and superior colliculus (SC). In particular, SC-projecting cdlSNr neurons respond automatically and differentially to stably high- and low-valued objects (Yasuda et al., 2012). They have long-term memories of object values (>100 days) with a high capacity (>300 experienced objects). These features are completely different from the rostral basal ganglia circuit, which does encode object values, but using short-term memories, and contributes to deliberate rather than automatic choice (Kim and Hikosaka, 2013). However, other brain areas may be involved. For example, neurons in the parietal cortex (LIP) retain their response biases based on previously over-learned reward values (Peck et al., 2009).

#### Reward uncertainty (risk)

Several cortical and subcortical areas contain neurons that encode reward uncertainty (McCoy and Platt, 2005; O'neill and Schultz, 2010; Monosov and Hikosaka, 2013). However, these data were obtained when the reward outcome was expected. The salience-related responses should be present regardless of the reward outcome, and this has not been examined. Furthermore, it is unknown whether and how these areas can control gaze (or eye movements) or attention. Uncertainty about outcome can also exist in the aversive domain (aversive risk). It remains to be seen whether and how such aversive risk influences aversive salience and interacts with various aversive outcomes.

#### Aversiveness

Neurons sensitive to aversive and threatening objects are found in many areas including the amygdala (Ledoux, 2000) and lateral habenula (Hikosaka, 2010). Again, the data were obtained when the outcome was expected. A feasible candidate for learned salience is dopamine neurons. Specifically, dopamine neurons in the dorso-lateral part of the substantia nigra pars compacta (SNc) in the monkey are excited by both rewarding and threatening (i.e., airpuff) objects (salience-type) (Matsumoto and Hikosaka, 2009). We recently found that dopamine neurons in this region of SNc project to CDt (Kim et al., 2014) and sustain their responses to high-valued objects even when reward is no longer expected (Kim et al., 2015). We hypothesize that the excitation of the salience-type dopamine neurons facilitates the CDt-cdlSNr-SC circuit so that gaze is attracted to both rewarding and threatening objects. In contrast, dopamine neurons in the ventro-medial part of SNc are excited by rewarding objects, but inhibited by threatening objects (value-type) (Matsumoto and Hikosaka, 2009). The value-type dopamine neurons may project to the head of the caudate nucleus (CDh; Kim et al., 2014), which is sensitive to the immediate value outcome (Kawagoe et al., 1998; Yasuda et al., 2012; Kim and Hikosaka, 2013; Kim et al., 2015). The threat-induced inhibition of the value-type dopamine neurons would suppress the CDh-circuit, so that during the choice trials the monkey tends not to choose objects that lead to threatening outcomes (Figure 5J). Furthermore, dopamine neurons differentially respond to airpuff and aversive tastants (Fiorillo et al., 2013). This may be related to the difference in salience between different kinds of aversiveness reported in our study.

#### Novelty

Neurons in many cortical and subcortical areas respond more strongly to novel objects than to familiar objects (Knight and Nakada, 1998; Ranganath and Rainer, 2003). Our results show a clear decrease in salience of novel objects by repeated exposure (Figures 7, 8) and by long-term familiarity (Figure 6). This might be executed simply by “habituation” for familiar objects with which signal transmissions of visual objects are depressed if repeated (Kemp and Manahan-Vaughan, 2004; Jutras and Buffalo, 2010). However, in our results, we tested for long-term effect of familiarity rather than recency. There is evidence for shared as well as dissociable encoding of recency vs. familiarity in the inferior temporal, perirhinal cortex. and hippocampus (Fahy et al., 1993; Charles et al., 2004). Whether negative salience of recent and familiar objects is mediated by the same or different neuronal mechanisms is not currently known and needs further investigation.

### Learned ecological salience: a defect or a basic skill for survival?

Positive or negative learned salience of objects can be distracting in certain conditions (Theeuwes and Belopolsky, 2012) and may contribute to mal-adaptive behaviors such as addiction (Robinson and Berridge, 1993; Field et al., 2006) or anxiety disorders (Bradley et al., 2000). However, in many situations in daily life, learned salience allows animals and humans to robustly adapt to their particular environment to find ecologically meaningful objects, accurately and quickly. This is done by guiding gaze to high-salience objects and rejecting gaze to low-salience objects. This benefit accumulates in life because emotional experiences of all objects are stored in a high capacity long-term memory. Without such an object-finding skill, the animal would fail to get rewards and avoid punishments, and may cease to survive (Hikosaka et al., 2013).

## Author contributions

AG and OH designed the experiments. AG and WG performed the experiments. AG analyzed the data with input from OH. All authors contributed to writing the paper.

### Conflict of interest statement

The authors declare that the research was conducted in the absence of any commercial or financial relationships that could be construed as a potential conflict of interest.
